# Case Report: A young man with mitochondrial disease: intellectual impairment and myocardial hypertrophy

**DOI:** 10.3389/fcvm.2026.1802202

**Published:** 2026-04-15

**Authors:** Xiao Luo, Ling Li, Gaofeng Su

**Affiliations:** 1 Department of Ultrasound, Affiliated Hospital of North Sichuan Medical College, Nanchong, China; 2 Department of Ultrasound, Suining Central Hospital, Suining, China

**Keywords:** cardiomyopathy, m.3243A > G variant, mitochondrial cardiomyopathy, mitochondrial diseases, MT-TL1 gene

## Abstract

Mitochondrial diseases are rare multisystem disorders caused by pathogenic variants in mitochondrial or nuclear DNA. We report a 23-year-old male presenting with exercise intolerance, fatigue, sluggish responsiveness, and a history of ptosis and bilateral hearing loss. Echocardiography revealed left ventricular hypertrophy, while brain MRI showed cerebellar atrophy and ventricular enlargement. Laboratory tests demonstrated elevated serum lactate, HbA1c, and high-sensitivity cardiac troponin T. Targeted sequencing identified a pathogenic m.3243A > G variant in the MT-TL1 gene with 55.6% heteroplasmy, confirming mitochondrial encephalomyopathy with cardiac involvement. This case highlights the phenotypic heterogeneity and diagnostic challenges of m.3243A > G–related disorders.

## Introduction

Mitochondrial diseases are a group of neuromuscular disorders caused by mutations in mitochondrial DNA (mtDNA) or nuclear DNA (nDNA), leading to impaired mitochondrial oxidative phosphorylation and ATP synthesis (1). Organs and tissues with high energy demands—such as the brain, myocardium, and skeletal muscles—are particularly affected (2). As a result, mitochondrial encephalomyopathy is a common manifestation of these conditions. Pathogenic mtDNA mutations impair myocardial glucose uptake, leading to disrupted cardiac energy metabolism and presenting as hypertrophic or dilated cardiomyopathy with conduction abnormalities (3). Involvement of skeletal muscle and the central nervous system may cause muscle weakness and atrophy, reduced exercise capacity, cognitive impairment, seizures, and headaches (4). Severe cases may develop respiratory distress. Cardiac involvement, especially myocardial hypertrophy, may represent an early or even predominant manifestation of mitochondrial disease. However, when presenting in isolation or with subtle extra-cardiac features, it may be easily misdiagnosed as primary hypertrophic cardiomyopathy, leading to delayed recognition of the underlying mitochondrial disorder.

Here, we report a young patient presenting with myocardial hypertrophy accompanied by neurological and muscular manifestations, who was ultimately diagnosed with mitochondrial encephalomyopathy with cardiac involvement after a prolonged diagnostic delay. This case highlights the importance of recognizing myocardial hypertrophy as a potential indicator of mitochondrial disease and underscores the risk of diagnostic delay in patients with atypical or multisystem presentations.

## Case presentation

A 23-year-old male was admitted to the hospital in 2025 due to ‘poor academic performance, language comprehension difficulties, exercise intolerance, and fatigue for several years’. The patient had a prior history of ptosis and underwent surgical treatment at another hospital in 2017. In 2018, he was noted to have a decline in hearing. There was no history of toxin exposure, infectious disease, or trauma. He was born at term following an uneventful pregnancy, and there was no family history of genetic disorders or consanguinity.

On physical examination, the patient was 166 cm tall and weighed 50 kg, with a body mass index (BMI) of 18.1. His blood pressure was within the normal range. Breath sounds were clear in both lungs, the cardiac rhythm was regular, heart sounds were normal, and no pathological murmurs were heard in any valve auscultation area. Neurological examination revealed impairment of higher cognitive functions, including decreased comprehension, memory, and calculation ability, without disorientation. In addition, the patient was uncooperative during the ocular movement examination; both eyes were in the primary position, and no ptosis was observed. There was no muscle atrophy or hypertrophy of the limbs, no fasciculations, and muscle tone was normal. Tendon reflexes in the lower limbs were not elicited. Bilateral finger-to-nose, heel-to-shin, and rapid alternating movements were normal. The Romberg test was negative, and bilateral pathological reflexes were also negative. No other significant abnormalities were noted.

**Figure 1:**
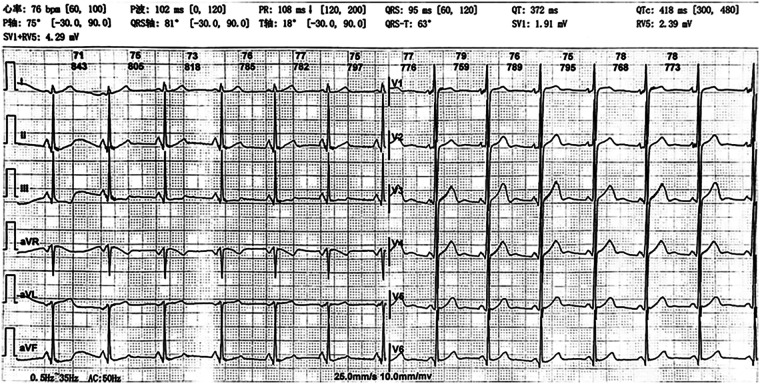
Electrocardiogram (ECG) findings. 1. sinus rhythm 2. shortened PR interval 3. Left ventricular high voltage.

**Figure 2:**
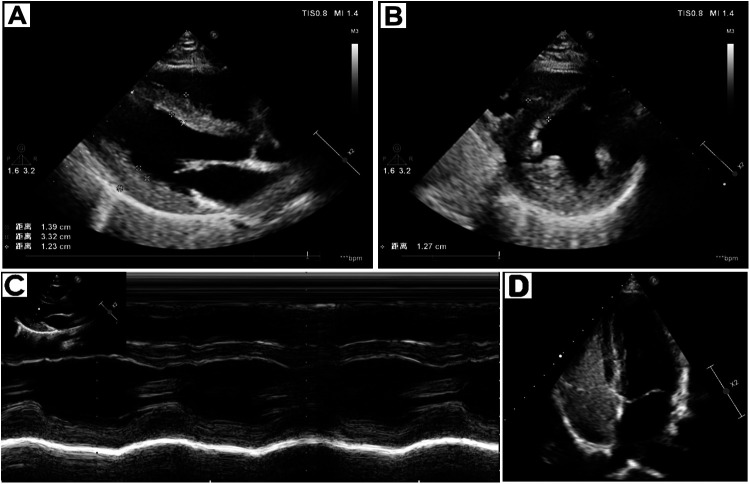
Echocardiogram funding. **(A)** Parasternal long-axis view of the left ventricle **(B)** Parasternal short-axis view at the level of the papillary muscles **(C)** M-mode echocardiogram of ventricular group **(D)** Right-Heart Contrast Echocardiography.

**Figure 3:**
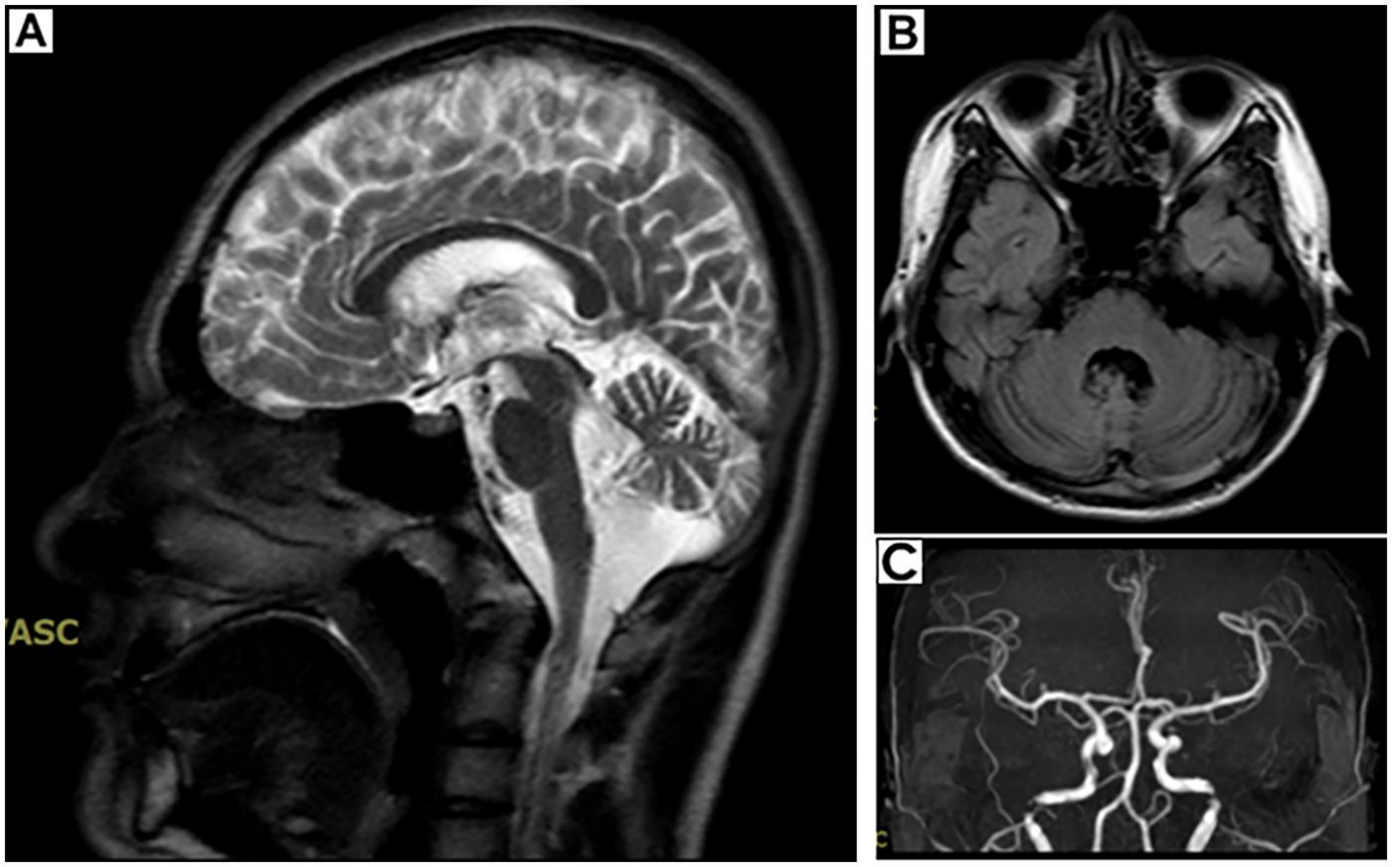
Head MRI. **(A)** Sagittal plane **(B)** Transverse plane **(C)** MRA.

**Figure 4:**
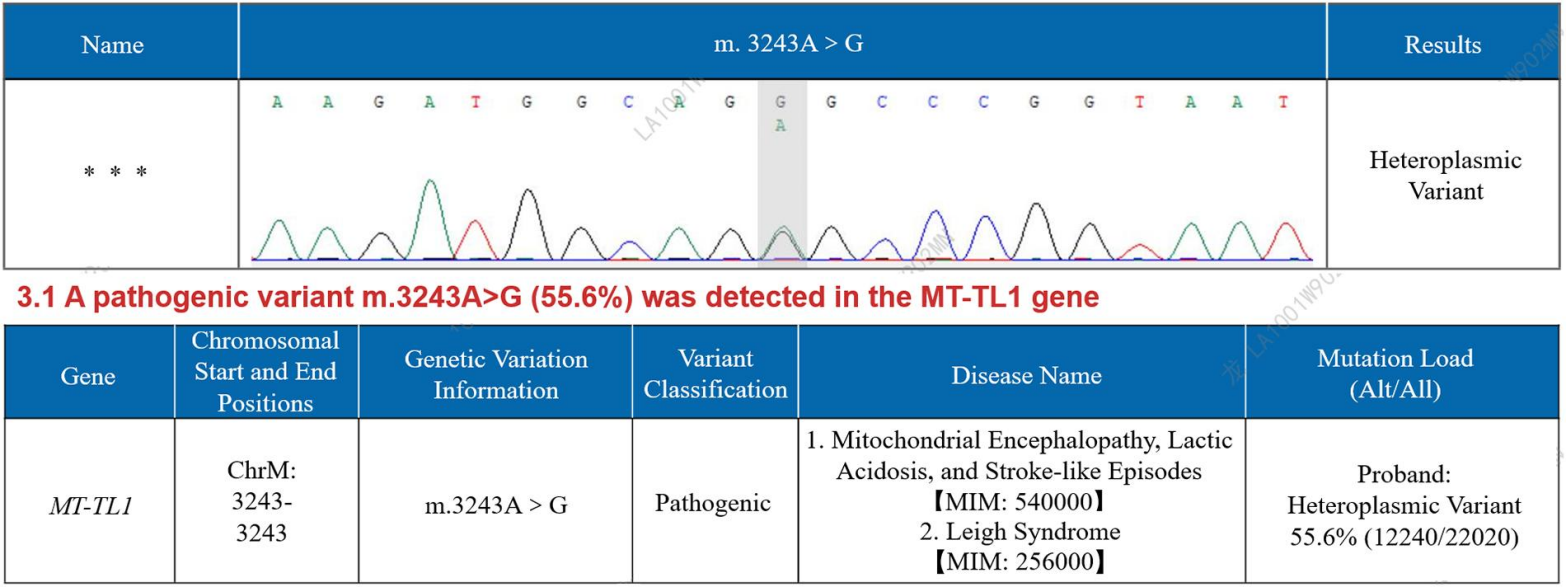
Genetic testing results of the patient.

Laboratory results showed the following: The levels of glycated hemoglobin (HbA1c), mean blood glucose (MBG), total glycated hemoglobin (GHb), high-sensitivity cardiac troponin T (hs-cTnT), erythropoietin (EPO), and lactate were elevated. The levels of hemoglobin (HGB), hematocrit (HCT), mean corpuscular volume (MCV), and mean corpuscular hemoglobin (MCH) were decreased (Table 1). The creatine kinase level and other laboratory test results were within the normal range (Supplementary Table S1). Electrocardiogram (ECG) revealed sinus rhythm, shortened PR interval, and left ventricular high voltage (Figure 1). Echocardiogram demonstrated left ventricular wall thickening, with interventricular septal thickness of approximately 12 mm and posterior wall thickness of 14 mm. (Figure 2). Dynamic images from the left ventricular long-axis view and the short-axis views at the level of the mitral valve and papillary muscles demonstrated left ventricular wall thickening more clearly (Supplementary Video S1–S4). Contrast echocardiography of the right heart revealed normal atrial septal anatomy with no evidence of atrial-level shunting (Supplementary Video S5). The patient's left ventricular ejection fraction (LVEF) was approximately 58%, which falls within the normal reference range; however, in a 23-year-old individual, this value can be considered at the lower limit of normal or slightly reduced. Based on the patient's clinical history, mitochondrial cardiomyopathy is highly likely. Cerebral MRI showed cerebellar atrophy and enlargement of the ventricular system, with notable enlargement of the fourth ventricle. Magnetic Resonance Angiography (MRA) revealed no signs of aneurysms or vascular malformations (Figure 3). MRI of the inner ear (including both plain and contrast-enhanced scans) showed no significant abnormalities in the bilateral inner ear structures, but possible softening lesions were noted in the bilateral corona radiata, along with evidence of brain atrophy and sinusitis. Cerebral angiography indicated that the morphology, course, and diameter of the cerebral vessels were generally normal. The Montreal Cognitive Assessment (MoCA) indicated moderate cognitive impairment, and the Mini-Mental State Examination (MMSE) also demonstrated cognitive dysfunction in the patient. Genetic testing of the patient's peripheral blood revealed a pathogenic m.3243A > G variant in the MT-TL1 gene, with a heteroplasmy level of 55.6% (Figure 4). Based on the patient's clinical manifestations, imaging findings, and genetic results, a comprehensive diagnosis of mitochondrial encephalomyopathy with cardiac involvement was established.

**Table 1:** Laboratory data.*

Variable	Result	Reference Range (Adults^†^)
**Blood Glucose Analysis**		
Glycated Hemoglobin (HbA1c)(%)	7.25↑	4.00–6.00
Mean Blood Glucose (MBG)(mmol/L)	10.06↑	3.60–7.60
Total Glycated Hemoglobin (GHB)(%)	9.43↑	3.90–7.30
**Blood**		
Hemoglobin (HGB) (g/L)	121↓	130–175
Hematocrit (HCT) 0.388↓ 0.400–0.500		
Mean Corpuscular Volume (MCV)(fl)	70.30↓	82.00–100.00
Mean Corpuscular Hemoglobin (MCH)(pg)	21.90↓	27.00–34.00
**Cardiac biomarkers**		
High-sensitivity Troponin T (hsTnt)(ng/L)	16↑	0–14
**Etiological analysis of anemia**		
Erythropoietin (EPO)(mIU/mL)	28.97↑	2.59–18.50
**Liver and Renal Function, Lipid Profile and Electrolytes**		
Aspartate Aminotransferase (AST)(U/L)	14↓	15–40
Lactate (Lact)(Mmol/L)	3.72↑	0.50–2.20

Positive laboratory test results.

The asterisk (*) indicates positive results in laboratory examinations; an upward arrow (↑) indicates that the measured value is higher than the normal reference range, and a downward arrow (↓) indicates that the measured value is lower than the normal reference range.

The patient received treatment with cytidine diphosphate choline, methylcobalamin, vitamin B1, coenzyme Q10, and adenosine triphosphate (ATP). Given that the patient's clinical symptoms were not severe and had minimal impact on their quality of life, the patient subsequently requested to be discharged.

## Discussion

In this case, the young patient presented with multisystem involvement, including central nervous system manifestations (cognitive impairment, cerebellar atrophy), skeletal muscle symptoms (reduced exercise tolerance), cardiac abnormalities (left ventricular hypertrophy), and metabolic disturbances (elevated glycated hemoglobin). When combined with the genetic testing results, the findings supported a diagnosis of mitochondrial encephalomyopathy with cardiac involvement. This case underscores the high heterogeneity and multisystemic nature of mitochondrial diseases, while also highlighting the diagnostic and therapeutic challenges faced in clinical practice.

During cardiac ultrasonography, the sonographer inquired about the patient's medical history and discovered a prior history of ptosis and corrective surgery. Additionally, the patient exhibited hearing impairment, diminished attention span, and cognitive decline. These multisystemic manifestations could not be solely explained by myocardial hypertrophy. Based on the patient's clinical history, electrocardiographic findings, and laboratory test results, the sonographer raised a strong suspicion of mitochondrial cardiomyopathy (MCM). MCM is characterized by structural or functional abnormalities of the myocardium resulting from genetic defects affecting the mitochondrial respiratory chain. It is not associated with coronary artery disease, valvular disorders, congenital heart disease, or hypertension (5). Clinical manifestations most frequently include hypertrophic cardiomyopathy, dilated cardiomyopathy, and left ventricular non-compaction (6). In clinical practice, the diagnosis of MCM can be considered when any of the following criteria are met: (i) the presence of respiratory chain enzyme deficiencies in muscle, fibroblasts, or platelets; (ii) characteristic histopathological features on muscle biopsy, such as ragged-red fibers on Gomori trichrome staining or reduced cytochrome c oxidase activity; (iii) ultrastructural evidence of marked mitochondrial proliferation or abnormal accumulation; (iv) the identification of mitochondrial DNA (mtDNA) mutations or deletions; or (v) a confirmed diagnosis of mitochondrial disease in a first-degree relative (7). In terms of differential diagnosis, the absence of a history of hypertension and the presence of a non-enlarged left atrium excluded hypertensive cardiomyopathy. Hypertrophic cardiomyopathy, which typically manifests as an isolated phenotype, was also inconsistent with the multisystem involvement observed in this case. Glycemic parameters indicated that the patient was at an early stage of diabetes, which does not support diabetes-related myocardial remodeling as the cause of ventricular wall thickening. Danon disease was likewise considered; however, it usually presents with more pronounced left ventricular hypertrophy and is generally not accompanied by hearing impairment, making it less compatible with this patient's clinical features (8). Notably, this patient exhibited elevated levels of HbA1c and MBG, indicating the presence of glucose metabolism abnormalities. This may be attributable to mitochondrial gene mutations leading to *β*-cell dysfunction in the pancreas (9). As the disease progresses, a definitive diagnosis of mitochondrial diabetes may become evident.

The current mainstay of treatment for mitochondrial diseases is “mitochondrial cocktail therapy”, which primarily involves supplementation with energy substrates such as coenzyme Q10 and ATP in order to enhance the function of the electron transport chain (10). However, this therapeutic approach has limited efficacy in reversing established structural damage, such as myocardial hypertrophy and cerebral atrophy. In the present case, the patient exhibited relatively mild symptoms and had not yet progressed to heart failure or severe neurological impairment. Nonetheless, given the progressive nature of mitochondrial disorders, future clinical deterioration remains a significant concern and warrants ongoing therapeutic intervention.

The hallmark of this case was the initial presentation with ptosis at age 15, for which corrective surgery was performed. Eight years later, echocardiography demonstrated left ventricular wall thickening, a non-enlarged left atrium, mildly reduced systolic function, and a shortened PR interval. Together with mild cognitive and auditory decline, these findings prompted strong suspicion of mitochondrial cardiomyopathy, subsequently confirmed by genetic testing. The patient thus experienced an eight-year diagnostic delay, during which the absence of timely recognition and intervention may have accelerated multisystem involvement. This case underscores the importance of considering mitochondrial disease in adolescents with even subtle skeletal muscle weakness and highlights the value of clinical and genetic screening of family members for early detection.

## Data Availability

The original contributions presented in the study are included in the article/Supplementary Material, further inquiries can be directed to the corresponding author.

## Supplementary material

Supplementary Video S1 shows left ventricular wall thickening in the long-axis view, without evident left atrial enlargement.

Supplementary Video S2 demonstrates normal opening and mobility of the mitral valve in the short-axis view.

Supplementary Video S3 reveals left ventricular wall thickening in the short-axis view at the papillary muscle level.

Supplementary Video S4 depicts an M-mode echocardiographic image at the papillary muscle level.

Supplementary Video S5 illustrates contrast echocardiography of the right heart, showing normal atrial septal anatomy without evidence of atrial-level shunting.
